# Low biological fluctuation of mitochondrial CpG and non-CpG methylation at the single-molecule level

**DOI:** 10.1038/s41598-021-87457-8

**Published:** 2021-04-13

**Authors:** Chloe Goldsmith, Jesús Rafael Rodríguez-Aguilera, Ines El-Rifai, Adrien Jarretier-Yuste, Valérie Hervieu, Olivier Raineteau, Pierre Saintigny, Victoria Chagoya de Sánchez, Robert Dante, Gabriel Ichim, Hector Hernandez-Vargas

**Affiliations:** 1 Department of Tumor Escape, Resistance and Immunity, TGF-Beta and Immuno-Regulation Team, Cancer Research Centre of Lyon (CRCL), INSERM U 1052, CNRS UMR 5286, UCBL1, Université de Lyon, Centre Léon Bérard, 28 rue Laennec, 69373 Lyon Cedex 08, France; 2 Department of Cellular Biology and Development, Instituto de Fisiología Celular, Universidad Nacional Autónoma de México (UNAM), Circuito Exterior s/n, Ciudad Universitaria, Coyoacán, 04510 Mexico City, Mexico; 3 Department of Surgical Pathology, Hospices Civils de Lyon, Groupement Hospitalier Est, Lyon, France; 4 Univ Lyon, Université Claude Bernard Lyon 1, INSERM, Stem Cell and Brain Research Institute U1208, Bron, France; 5 Univ Lyon, Université Claude Bernard Lyon 1, INSERM 1052, CNRS 5286, Centre Léon Bérard, Centre de Recherche en Cancérologie de Lyon, Lyon, France; 6 Department of Translational Medicine, Centre Léon Bérard, Lyon, France; 7 Dependence Receptors Cancer and Development Laboratory, Department of Signaling of Tumoral Escape. Cancer Research. Center of Lyon (CRCL), Inserm U 1052, CNRS UMR 5286, Université de Lyon, Centre Léon Bérard, 28 rue Laennec, 69373 Lyon Cedex 08, France; 8 Cancer Cell Death Laboratory, Part of LabEx DEVweCAN, Université de Lyon, Lyon, France; 9 Cancer Research Centre of Lyon (CRCL), Inserm U 1052, CNRS UMR 5286, Université de Lyon, Centre Léon Bérard, 28 rue Laennec, 69373 Lyon Cedex 08, France

**Keywords:** Epigenomics, Cell biology, Genomics

## Abstract

Mammalian cytosine DNA methylation (5mC) is associated with the integrity of the genome and the transcriptional status of nuclear DNA. Due to technical limitations, it has been less clear if mitochondrial DNA (mtDNA) is methylated and whether 5mC has a regulatory role in this context. Here, we used bisulfite-independent single-molecule sequencing of native human and mouse DNA to study mitochondrial 5mC across different biological conditions. We first validated the ability of long-read nanopore sequencing to detect 5mC in CpG (5mCpG) and non-CpG (5mCpH) context in nuclear DNA at expected genomic locations (i.e. promoters, gene bodies, enhancers, and cell type-specific transcription factor binding sites). Next, using high coverage nanopore sequencing we found low levels of mtDNA CpG and CpH methylation (with several exceptions) and little variation across biological processes: differentiation, oxidative stress, and cancer. 5mCpG and 5mCpH were overall higher in tissues compared to cell lines, with small additional variation between cell lines of different origin. Despite general low levels, global and single-base differences were found in cancer tissues compared to their adjacent counterparts, in particular for 5mCpG. In conclusion, nanopore sequencing is a useful tool for the detection of modified DNA bases on mitochondria that avoid the biases introduced by bisulfite and PCR amplification. Enhanced nanopore basecalling models will provide further resolution on the small size effects detected here, as well as rule out the presence of other DNA modifications such as oxidized forms of 5mC.

## Introduction

It has long been established that mitochondria are the powerhouse of our cells. They are responsible for producing ATP through the electron transport chain, contributing to the cellular energetic and redox homeostasis^1^. In addition, mitochondria have diverse functions that include the regulation of apoptotic pathways as well as storing calcium for cell signaling^1^. The number of mitochondria in a single cell can vary widely; some cells having no mitochondria, such as red blood cells, with other cells having hundreds, such as liver cells^2^. Mitochondrial DNA (mtDNA) in humans has a molecular length of 16.5 kb and is comprised of a Heavy Strand (HS) and a Light Strand (LS), with an absence of histones and particular DNA repair requirements^3^. This unique biology leaves mtDNA exposed to influencing factors from both intra- and extra-cellular origin. For example, reactive oxygen species can increase mtDNA copy number^4^ and exposure to chemicals can cause mtDNA damage^5^. Moreover, alcohol exposure can induce oxidative stress^6^ and increase the expression of mtDNA methyl transferases (mtDNMT1)^7^. These events highlight the sensitivity of mitochondria to environmental factors which can have downstream consequences for cellular respiration as well as cancer development and progression.

Regulation of mtDNA gene expression occurs primarily through the Displacement loop (D-loop), a 1200-bp non-coding region of the mitochondrial genome. This region controls mitochondrial replication as well as transcription of its encoded genes through a number of different start sites and promoter regions^8,9^. Among regulatory mechanisms in nuclear DNA, DNA methylation is well characterized and known to be influenced by metabolic activity. In the human genome, cytosine methylation (5mC) occurs mainly in a CpG context (i.e. a cytosine followed by a guanine). In contrast, the existence of mitochondrial cytosine methylation has been a topic of debate, with evidence for high levels of mtDNA 5mC in certain human cells and strand-biased non-CpG (CpH) methylation^7,10–12^. However, other studies suggested that some of these findings were due to incomplete bisulfite conversion being caused by a failure to linearize mtDNA prior to sequencing^13–15^. Moreover, the tools to analyse the presence of DNA methylation rely heavily on sodium bisulfite conversion and PCR amplification; which damage DNA and can lead to strand- or sequence-dependent biases^16^.

Nanopore sequencing is a unique, scalable technology that enables direct, real-time analysis of long DNA or RNA fragments^17,18^. It works by monitoring changes to an electrical current as nucleic acids are passed through a protein nanopore. The resulting signal is decoded to provide the specific DNA or RNA sequence. Moreover, this technology allows for the simultaneous detection of nucleotide sequence and DNA and RNA base modifications on native molecules^19^; hence, avoiding introduced bias from sodium bisulfite treatment and PCR amplification.

The overall aim of this study was to shed light into the presence or absence of mtDNA 5mC methylation (5mCpG and 5mCpH) using a base-resolution technique that overcomes the limitations of bisulfite sequencing. In addition, we aimed to determine 5mC variation across different biological conditions. Three cellular settings known to influence mitochondrial dynamics were analyzed: cellular differentiation, oxidative stress and cancer. Both CpG and nonCpG methylation levels were low and unchangeable across all contexts, with some exceptions related to cell culture and malignancy.

## Results

### Nanopore sequencing reliably detects CpG DNA methylation in CpG context (5mCpG)

**Figure 1:**
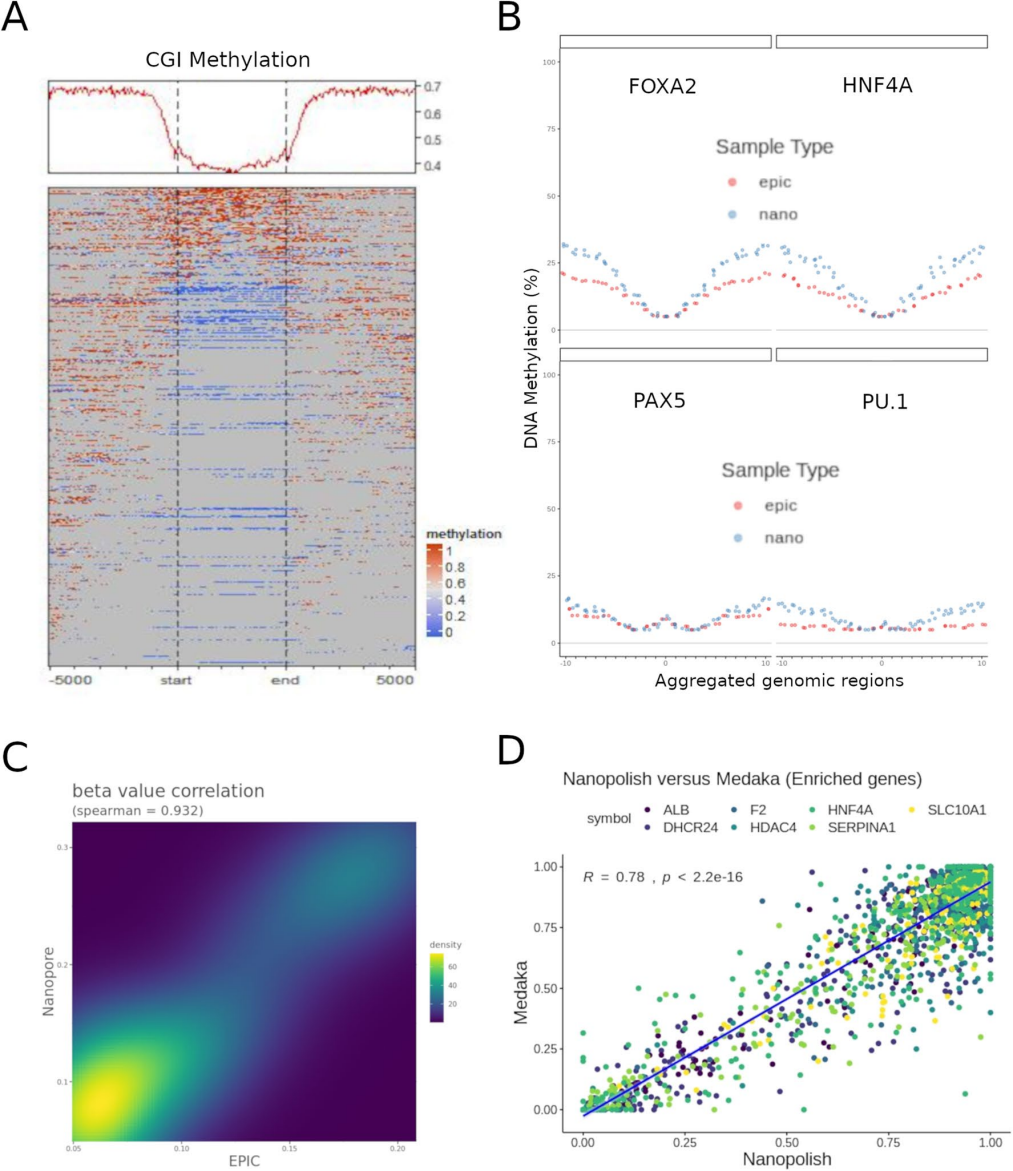
Inspection of 5mCpG data obtained using nanopore sequencing. (**A**) Global profile of nuclear DNA methylation at CpG islands (CGI), obtained after nanopore sequencing of the human liver cell line HepaRG. (**B**) Aggregated DNA methylation data was obtained for hepatocyte active (FOXA2 and HNF4A, top panels) and control (PAX5 and PU.1, bottom panels) transcription factor binding regions. Methylation profiles from EPIC bead array (epic) and Nanopore sequencing data (nano) (red and blue lines, respectively) are shown for each aggregated dataset. (**C**) EPIC-Nanopore correlation for 5mCpG data on all aggregated datasets shown in (**B**). (**D**) Nanopore targeted sequencing for a panel of hepatocyte identity genes was used to basecall 5mCpG using two different bioinformatic pipelines: Nanopolish and Guppy + Medaka (see “Methods”). Single CpG level correlations are shown.

Long read sequencing is a rapidly evolving field that is largely still in its infancy. Hence, we first sought to determine the reliability of using nanopore sequencing to detect DNA methylation from native DNA in our own hands. To do so, we sequenced genomic DNA extracted from the human liver cell line HepaRG, using an Oxford Nanopore Minion device (ONT). Global patterns of DNA methylation were consistent with the known depletion of 5mCpG at CpG islands (CGIs) (Fig. 1A). We then compared genome-wide methylation patterns of DNA from HepaRG cells sequenced with nanopore, to those obtained with EPIC Bead Arrays (Illumina)^20^. To overcome the problem of sparsity in DNA methylation data, we aggregated CpG methylation values from more than 130 k transcription binding site loci corresponding to hepatocyte-specific (FOXA2 and HNF4A) and control (PAX5 and PU.1) target regions. Both, EPIC and Nanopore data are able to capture the expected dip in methylation associated with active regulatory regions (Fig. 1B, top panels)^21^. In contrast, non-active transcription factor binding sites produce a flat methylation profile after aggregation of a similar number of genomic regions in both EPIC and Nanopore data (Fig. 1B, bottom panels). Both techniques were highly correlated when aggregated data from all transcription factor binding sites was taken together (Fig. 1C).

Different methods have been described for extracting base modification information from nanopore sequencing data, and recently they have been carefully benchmarked^22^. In line with recommendations, we tested two of the best established tools to detect CpG methylation, Nanopolish^23^ which uses a hidden markov model to detect DNA methylation, and the more recent Guppy + Medaka which has been trained to basecall for modified human CpG dinucleotides using a recurrent neural network^24^. According to the benchmarking described above, while Guppy correctly identified unmethylated sites, Nanopolish was better at recovering fully methylated sites^22^. To perform a site-level correlation, we used targeted nanopore sequencing data from HepaRG cells with a higher coverage in a set of hepatocyte identity genes (i.e. *ALB, F2, HNF4A, SLC10A1, DHCR24, HDAC4* and *SERPINA1*). Using this method of comparison, DNA methylation values were highly correlated, with Guppy + Medaka having a higher tendency towards calling cytosines as unmethylated (Fig. 1D). These results are in line with former studies showing a slightly higher tendency for Medaka to call unmethylated cytosines^25^, and Guppy to underpredict high methylation compared to Nanopolish^22^.

Therefore, in agreement with recent publications, 5mCpG methylation can be reliably obtained from native DNA using nanopore sequencing and different bioinformatic algorithms. For all CpG analyses presented below we used Guppy + Medaka for extraction of 5mCpG values and Nanopolish for verification and visualization.

### Detection of mtDNA 5mCpG in long reads

**Figure 2:**
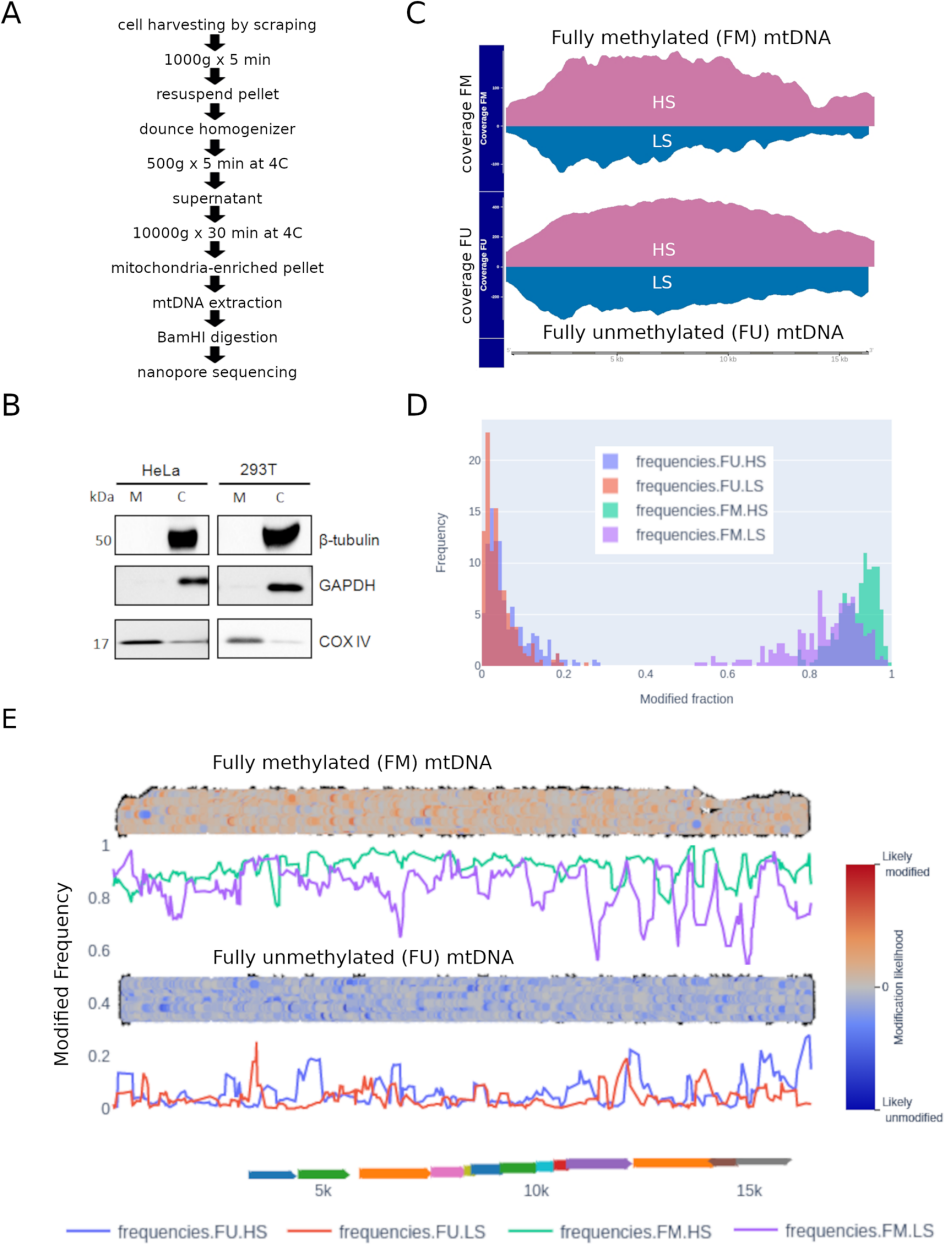
5mCpG methylation in mtDNA. (**A**) Protocol of subcellular fractionation and mtDNA extraction used before nanopore sequencing. (**B**) Quality control of mtDNA enrichment in different cell lines (i.e. HeLa and 293 T) using western blot against b-Tubulin, GAPDH, and COX-IV in mitochondrial (M) and cytosolic (C) fractions (supplementary Figure S1). (**C**) The same protocol was used on the liver cell line HepaRG. In addition, whole genome amplification was used to produce a “fully” unmethylated control (FU), and followed by DNA methylase (M.Sssl) treatment to produce a “fully” methylated control (FM). Nanopore sequencing coverage for the heavy strand (HS) and the light strand (LS) in FU and FM mtDNA-enriched HepaRG samples. (**D**) Nanopolish was used to infer 5mCpG likelihood and extract methylation frequency tables (corresponding to the proportion of methylation). Methplotlib (see “Methods”) was used to plot a histogram of 5mCpG frequencies in FU and FM, colored by strand. (**E**) Strand-specific 5mCpG frequency plots (colored lines), and 5mCpG likelihood pile-plots (100 reads per sample). Gene mapping to mtDNA are shown in the bottom track as colored arrows. “Modified frequency” refers to the proportion of modified base calls, in this case 5mCpG, for each of the samples and CpG sites.

Having shown the suitability of nanopore sequencing for analysis of nuclear 5mCpG, we used the same strategy on mtDNA enriched by subcellular fractionation of different cell lines (Fig. 2A). Importantly, mtDNA was linearized enzymatically before sequencing using a Minion device. This technique enabled the clear enrichment of the mitochondrial cellular fraction, measured by protein expression of mitochondrial or cytosolic markers (Fig. 2B and Supplementary Figure S1). After sequencing, we obtained a high fraction of reads mapping to mtDNA. Of note, due to long read length, the proportion of mapped reads and their coverage was higher than 80%. Indeed, some reads consisted of full-length mtDNA sequences (Fig. 2C). Interestingly, we observed an unequal representation of the heavy strand (HS) and light strand (LS) of mtDNA (Fig. 2C). We attributed this to the efficiency of the BamHI enzyme in its ability to cut the HS more efficiently and leaving a slightly higher ratio of 5′ ends available for the ligation of adapters before loading onto the Nanopore sequencing device. Moreover, recent findings suggest there can be an unequal representation of mtDNA CpG methylation on the HS and LS^10^. Hence, to reduce any potential bias to the average methylation of each CpG site, we considered the methylation of the HS and the LS separately for further analysis. Furthermore, mitochondrial populations can be heterogeneous within a single cell. Therefore, we also took advantage of single molecule methylation, by visualizing the methylation of whole mtDNA reads to better understand the single molecule methylation heterogeneity in our samples.

To validate the accuracy of nanopore for detecting 5mCpG, we prepared fully unmethylated (FU) and fully methylated (FM) mtDNA controls. FU was prepared by whole genome amplification and then FM was prepared by methylation of CpG nucleotides using DNA methyltransferase (M.Sssl). As expected, 5mCpG profiles were opposite in FU and FM mtDNA controls (Fig. 2C,D). Some residual methylation was observed in the FU control and we considered these levels as a baseline for this technique (Fig. 2D,E). Indeed, we used the FU control as our background to call detectable methylation. This value was obtained by dividing the number of called sites as methylated by the total number of called sites in the FU sample. The background calculated for the mtDNA HS was 0.022 and for the LS 0.016. We were also able to identify some fully unmethylated reads in the FM control. We attributed this to incomplete efficiency of the DNA methyltransferase (M.Sssl) in methylating these reads, and therefore a stochastic effect. Furthermore, this observation highlights the utility of this approach to identify a mixture of DNA in a single sample. In addition, we observed low basal levels of 5mCpG in mtDNA from HepaRG cells. Indeed, we did not identify any differential methylation between the FU control and HepaRG cells, either globally or at the CpG site or strand-specific levels (see next Section).

These data shows that Nanopore sequencing is able to detect mtDNA methylation above an acceptable background. 5mCpG is not different from the unmethylated control at HepaRG basal conditions. We next went further to investigate 5mCpG in several conditions known to modify mitochondrial activity.

### mtDNA CpG methylation was not affected by in vitro hepatocyte differentiation

**Figure 3:**
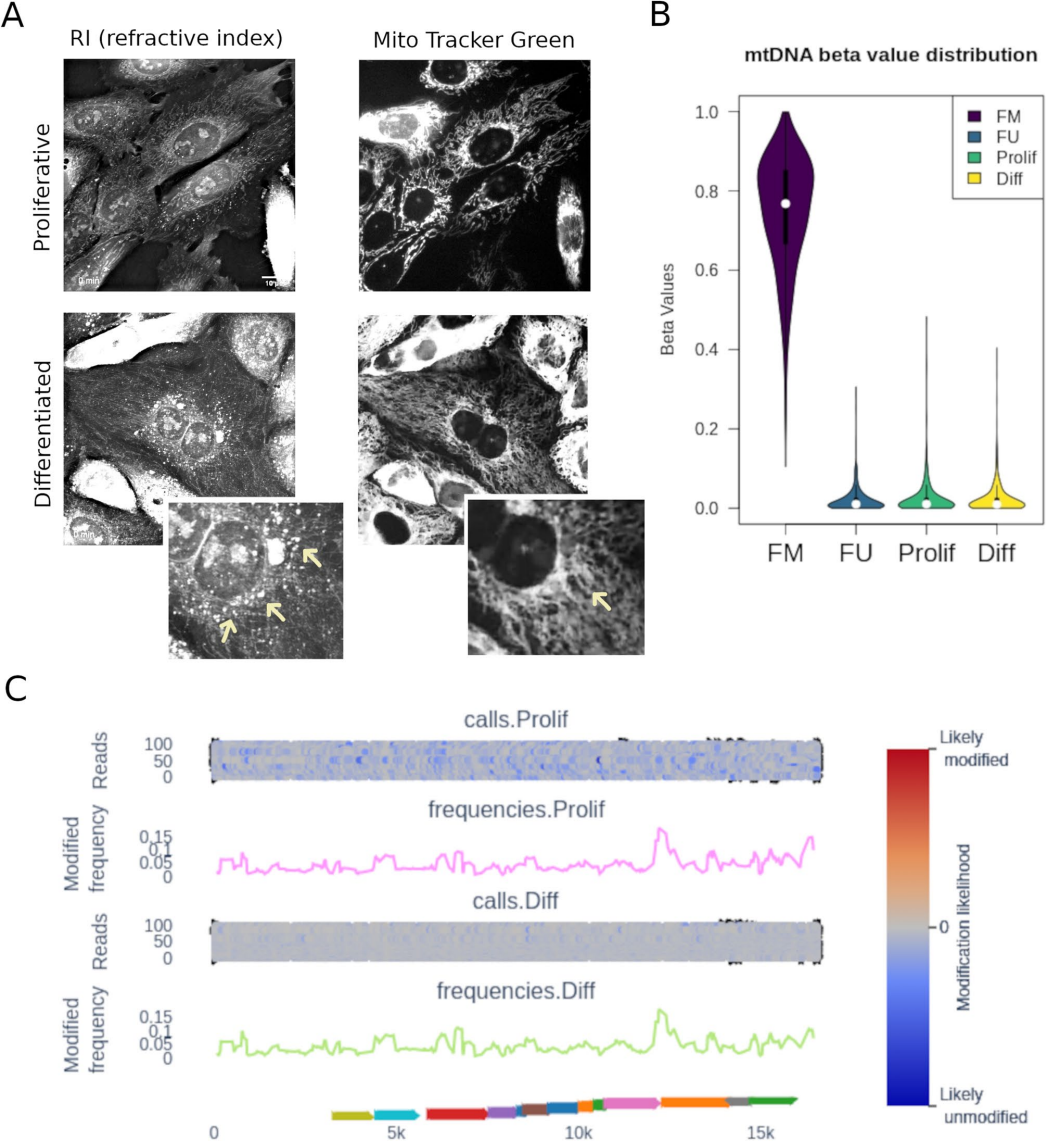
Methylation of mitochondrial DNA measured by nanopore sequencing of a liver progenitor cell line. (**A**) Holotomography images of proliferative (progenitor) HepaRG cells and their differentiated progeny. Left panel: Refractive Index (RI) map. Right panel: MitoTracker Green staining to distinguish mitochondrial content and distribution. Insets with higher magnification are shown for differentiated HepaRG, with arrows indicating lipidic droplets and denser mitochondrial network in RI and Mito Tracker Green, respectively. (**B**) mtDNA enriched DNA extracts from HepaRG cells were linearized and sequenced, as described in Fig. 2A. The distribution of 5mCpG beta values (proportion of methylated calls relative to the total calls for a given site) in both strands combined is shown for proliferative (Prolif) and differentiated (Diff) HepaRG, as well as FM and FU controls. (**C**) Methylation frequency and likelihood (pile-plots for the first 100 reads) is shown for proliferative and differentiated HepaRG (one representative sample of three independent differentiation assays). Methylation likelihood scale shown in the pile-plots represents unlikely methylated in blue, likely methylated in red, and intermediate values in gray. Gene mapping to mtDNA are shown in the bottom track as colored arrows.

Hepatocyte differentiation implies metabolic rewiring and changes in mitochondrial content and activity^26^. As such, this dynamic process may involve concomitant changes in mtDNA methylation. The bipotent liver progenitor cells, HepaRG, are capable of in vitro differentiation into hepatocytes and biliary cells. By plating HepaRG cells under differentiating conditions during four weeks we obtain a mixture of the hepatocyte and biliary lineages^20,27,28^. This well-established model allows us to compare hepatic “progenitor like” cells to their “differentiated” counterpart. We used minimally photo-toxic holo-tomographic microscopy combined with mitochondrial labelling (using MitoTracker Green) to determine mitochondrial content. We observed in both cellular tomogram and MitoTracker staining profile that differentiated HepaRG cells have a higher mitochondrial content, as well as more lipid droplets when compared to their progenitors (Fig. 3A).

To determine the effect of hepatocyte differentiation on mtDNA methylation, we used the nanopore sequencing protocol described above, comparing progenitor-like HepaRG cells with their differentiated progeny. In both cases, methylation values were not different from the fully unmethylated control (Fig. 3B). There was no differential methylation when directly comparing proliferative and differentiated HepaRG cells (Fig. 3C). Similar results were obtained when analyzing both strands together or independently. Interestingly, the likelihood of methylation, as calculated with nanopolish, was higher in differentiated HepaRG cells. This can be seen at the read level (likelihood scale in Fig. 3C shows mainly blue reads in proliferative and mainly gray reads in differentiated cells), although this difference was not high enough to be called as methylation and/or may represent additional nucleotide modifications.

### mtDNA CpG methylation does not fluctuate under in vitro oxidative stress conditions

**Figure 4:**
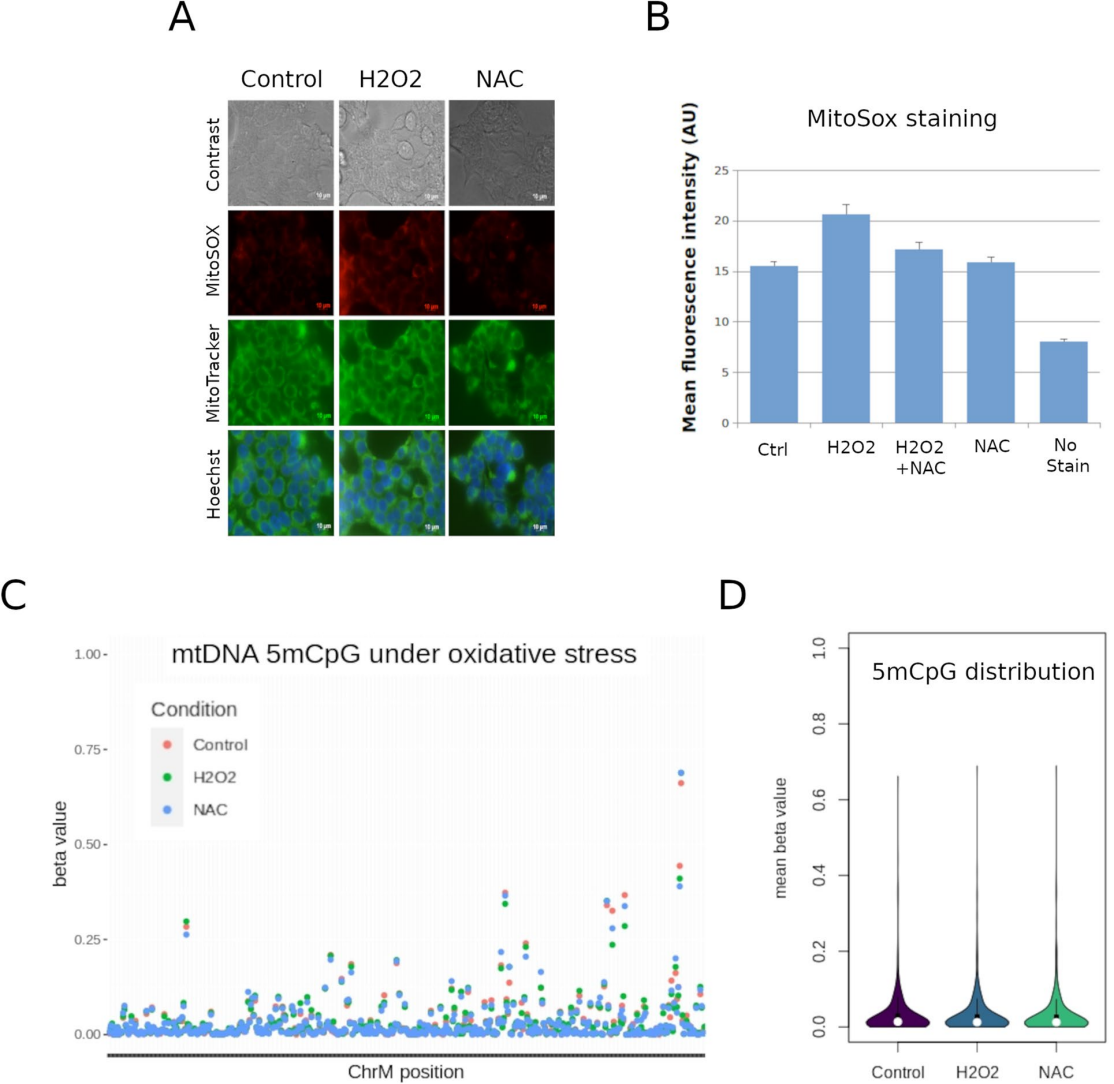
Mitochondrial 5mCpG in response to oxidative stress. Mitochondrial DNA was obtained from the human embryonic kidney cell line 293 T under basal conditions (Control), oxidative stress (H2O2) and oxidative stress rescued with N-acetlycysteine (NAC). (**A**) Representative images of phase contrast, Mitosox, MitoTracker and Hoescht staining. (**B**) MitoSox quantification of 5 independent replicates. (**C**) 5mCpG measured by nanopore under the same experimental conditions. Each dot represents the average of triplicate values for each condition and each CpG site. (**D**) Mitochondrial 5mCpG distribution (both, HS and LS strands together) of the data shown in (**C**).

In addition to differentiation, mitochondrial activity is largely associated to oxidative stress, and therefore an interesting process where to study 5mCpG variation. To induce oxidative stress in vitro, we used an established method utilizing hydrogen peroxide (H2O2) to induce reactive oxygen species^29^. Several cell lines were tested (data not shown) and *Homo sapiens* embryonic kidney 293 T cells emerged as an ideal candidate for an oxidative stress model. Treatment for two hours was sufficient to induce oxidative stress in 293 T cells as measured by MitoSox staining, which could be rescued by treatment with N-acetylcysteine (NAC) (Fig. 4A,B).

At basal levels, 293 T cells exhibited higher global levels of 5mCpG than HepaRG cells (p value = 0.05). However, the same strand specific methylation was observed (i.e. higher 5mCpG in the HS). We then compared 5mCpG levels in control, H2O2-treated, and NAC-rescued 293 T cells. There were no global differences in mean methylation between groups, and with a threshold of 10%, we did not observe any differential methylation on the HS or the LS at the single CpG level (Fig. 4C,D).

Although 5mCpG was clearly detectable in 293 T cells, and higher than HepaRG cells at steady state, it was not significantly affected by H2O2 exposure.

### Effect of cell culture on CpG methylation

**Figure 5:**
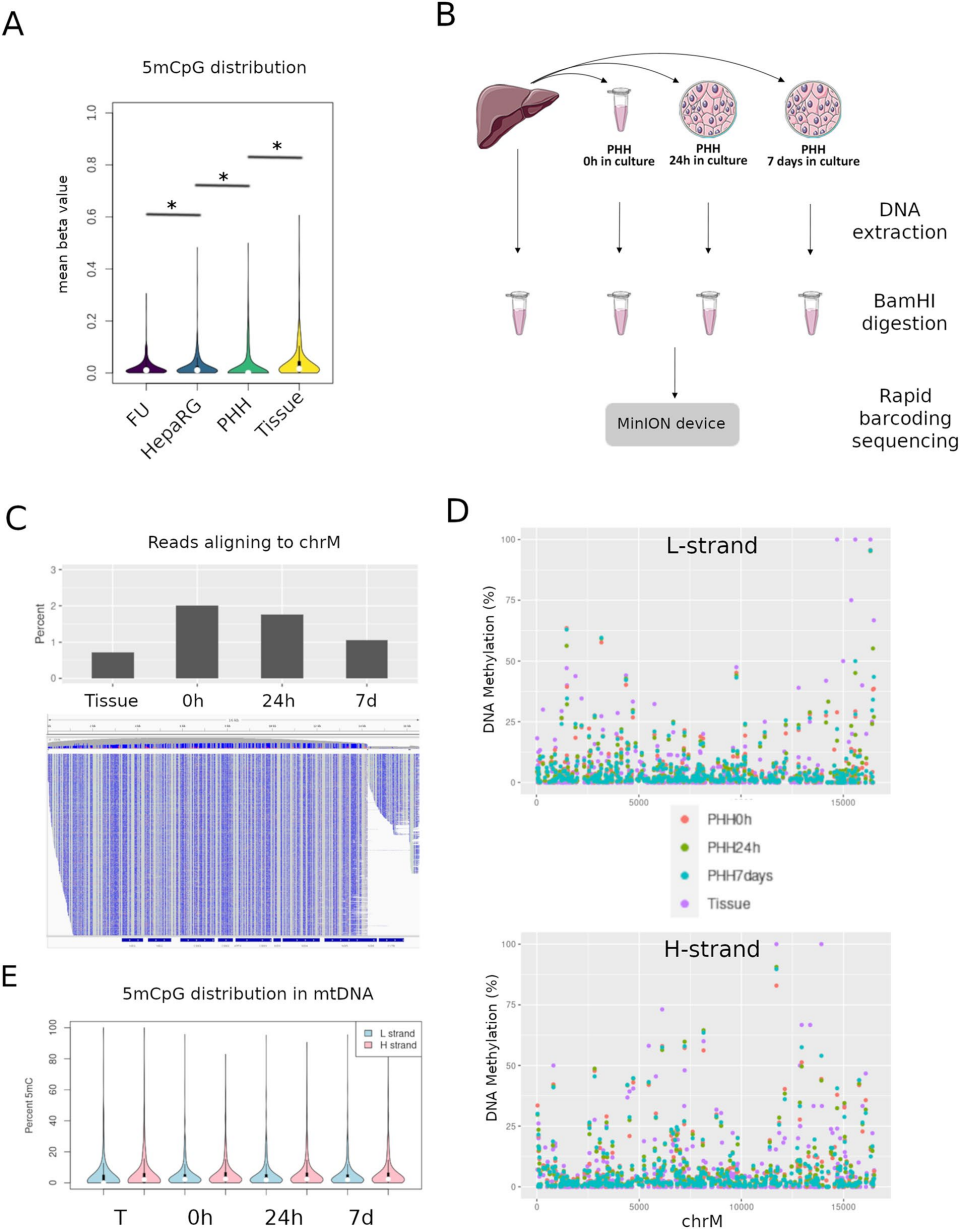
Hepatocyte methylation upon cell culture. (**A**) 5mCpG distribution along mtDNA for fully unmethylated control (FU), proliferative HepaRG cells, primary human hepatocytes (PHH) and one representative non-tumor liver tissue (Tissue). (*) indicates p value < 0.05, Mann–Whitney’ test. (**B**) mtDNA DNA methylation after hepatocyte isolation and culture conditions in a kinetics experiment. (**B**) Experimental overview, PHH were isolated form healthy liver tissue and cultured for 0 h, 24 h or 7 days before DNA extraction and sequencing with Nanopore. (**C**) Percentage of reads aligning to the chrM for each sample (upper panel), and visualization of aligned reads to chrM in IGV (lower panel), reads converted to identify 5mCpG (blue = unmethylated cytosine, red = methylated cytosine). (**D**) Base level 5mCpG on L-strand (upper panel) and H-strand (lower panel) in each sample. (**E**) 5mCpG distribution of L and H strands between each sample and time point.

Global differences in DNA methylation have been described after cells are plated in culture conditions^30^. Moreover, major metabolic alterations, notably metabolic repression, have been described after hepatocytes are placed in culture (Cassim et al., 2017). While the liver cell line HepaRG did not display 5mCpG above background (Fig. 3), human liver tissues were consistently methylated at discrete CpG sites and globally more methylated (Fig. 5A). In line with this, we observed intermediate 5mCpG values in primary human hepatocytes (PHH) after two weeks in culture (Fig. 5A). Globally, 5mCpG was not different in HepaRG as compared to the FU control (p = 0.5). In contrast, 5mCpG was higher in PHH relative to HepaRG (p < 2.2e−16), and higher in liver tissues relative to PHH (p < 2.2e−16) (Fig. 5E). This result was similar when analyzing separately both mtDNA strands.

The results above suggest that 5mCpG may be lost in culture conditions. To systematically evaluate the effect of cell isolation and culture on mtDNA methylation under controlled conditions, we obtained healthy liver tissue and isolated PHH from the same sample (Fig. 5B). DNA was extracted from liver tissue, PHH in suspension (0 h) and also placed in culture for 24 h or 7 days before isolation of DNA, enzymatic linearization and sequencing with Nanopore’s MinION (Fig. 5B). Despite omitting the mtDNA enrichment step, between 1–2% of reads aligned to the mitochondrial chromosome, representing coverage of over 1000 × for each condition (Fig. 5C). Visualization of 5mCpG at single base resolution revealed slightly higher levels in liver tissue compared to PHH cultured for 7 days on the L-strand and the H-strand (Fig. 5D,E). However, we did not observe any differences between PHH 0 h in culture and 7 days.

Thus, 5mCpG differences between liver tissue and cultured PHH are more likely due to the combination of cells present in tissue rather than culture conditions. While levels of 5mC were low in mtDNA, we were confident in these findings after validating with an additional BS-qMSP technique (Supplementary Figure S2).

### Nanopore sequencing detects low CpH mtDNA methylation across diverse biological conditions

**Figure 6:**
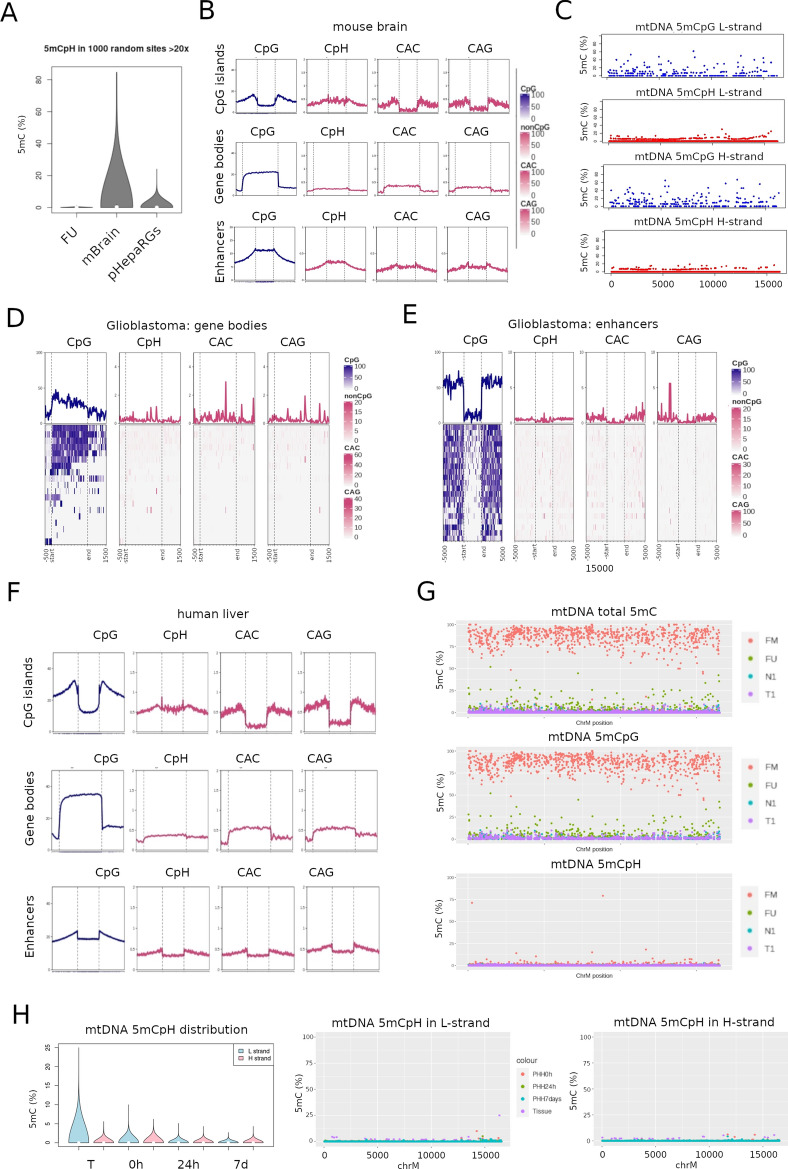
NonCpG methylation using nanopore single molecule sequencing. Raw data (fast5) from different samples and conditions was basecalled using an all-context 5mC neural network model. (**A**) 5mC distribution in non-CpG (CpH) context comparing fully unmethylated control (FU), one mouse brain sample (mBrain) and the proliferative HepaRG cell line (pHepaRG). (**B**) Total (nuclear DNA) 5mC information from the mouse brain sample was aggregated to create enriched heatmaps according to mm10 annotated CpG islands, gene bodies and enhancer regions. Average methylation plots are shown for CpG, CpH, or specifically for CAC and CAG context. (**C**) Mitochondrial DNA methylation (% 5mCpG) in the same mouse brain sample, presented by strand (L-strand : top plots, H-strand: bottom plots) and cytosine context (CpG: blue, CpH: red). (**D**) Similar analysis was done in cas9-targeted data of human glioblastoma cell lines and tissues^53^. Combined 5mC data (6 human diffuse gliomas and 3 human glioblastoma cell lines) was aggregated according to (**D**) gene bodies, and (**E**) enhancers. (**F**) Similar to (**A**), we performed all context 5mC base calling in a human liver sample, and aggregated the data according to genomic context. (**G**) The same liver sample shown in (**F**) was used to extract reads aligning to mtDNA (N1). A matched tumor sample (T1) and FM/FU controls are also shown in separate plots for total 5mC (top panel), 5mCpG (middle panel) and 5mCpH (bottom panel). (**H**) 5mCpH values corresponding to the PHH kinetic experiment described in Fig. 5. Genomic 5mCpH distribution is shown in (**H**) and mtDNA 5mCpH is shown in (**I**) separately for the L- (left panel) and H- (right panel) strands.

In addition to 5mCpG, new algorithms have become recently available for the assessment of 5mC in non-CpG (CpH) contexts from nanopore sequencing data. These algorithms are based on training with reference sequences using deep learning and are contained in the Rerio nanopore repository (ONT). Once the model is defined, a research command line tool (i.e. Megalodon, see “Methods”) is used to extract high accuracy modified base from raw nanopore reads by anchoring the basecalling output to a reference genome. From the Rerio repository, we selected the most recent version trained to detect all-context 5mC on MinION raw sequencing data (see “Methods”). To validate the performance of this algorithm for CpG and CpH 5mC, we first used nuclear DNA extracted from central nervous tissues, known to be relatively abundant in 5mCpH in physiological conditions. 5mC was overall higher in mouse brain, compared to the cell line levels described above (Wilcoxon rank sum test p-value < 2.2e−16) (Fig. 6A). Mouse brain DNA displayed the expected genomic 5mCpG distribution of absence in CpG islands, enrichment in gene bodies and intermediate levels in distal enhancers (Fig. 6B). As previously described, CpH methylation followed this same global pattern with particular enrichment (up to 5% methylation) in gene bodies, although we did not observe an obvious bias towards CAC versus CAG context (Fig. 6B)^31–33^. Even low coverage sequencing obtained with Cas9-targeting followed by nanopore sequencing, was able to show gene body CpH methylation in a panel of human glioblastoma cell lines and diffuse glioma tissues (Fig. 6D,E). Similarly, CpH was absent or low in CpG islands (CGI) and enhancers.

Next, we used the same algorithm to extract base modification information from mouse brain mtDNA. Interestingly, 5mCpG was detectable above background levels for both, L- and H- strands (Fig. 6C). In contrast, most cytosines in CpH context displayed levels below the threshold of detection in both mtDNA strands (Fig. 6C). We were also able to detect the expected patterns of nuclear CpG and CpH methylation in human liver tissues using this all-context model (Fig. 6F). However, both CpG and CpH were detected at low levels in mtDNA, confirming our previous findings with different algorithms (i.e. Nanopolish and Guppy + Medaka) (Fig. 6G).

Our results for mtDNA 5mCpG using this new model confirmed our previous results obtained in differentiation and oxidative stress cell line models. In addition, CpH levels were low and stable across all conditions. Interestingly, in the PHH time-point experiment, despite the low levels of non-CpG methylation in mtDNA, liver tissue had higher levels of this modified base compared to PHH in culture for > 24 h (Fig. 6H,I), similar to what we observed for 5mCpG, and suggesting that cell plating results in loss of 5mCpN in general. Low 5mC levels were validated by qMSP (Supplementary Figure S2).

In summary, new deep learning algorithms for calling base modifications confirm our results for mtDNA 5mCpG. In addition, they are useful in detecting expected nuclear CpH methylation and suggest general low levels and fluctuation in mtDNA 5mCpH.

### mtDNA methylation in cancer

Most cancer cells undergo a switch in their metabolic configuration, primarily relying on aerobic glycolysis instead of mitochondrial oxidative phosphorylation^34^. In addition, it was recently described that mtDNA from liver cancer cells had higher levels of CpG and nonCpG methylation than that of non-tumorigenic liver cells in vitro^35^. With this in mind, we performed whole genome nanopore sequencing of DNA from matched tumor/normal samples of liver (hepatocellular carcinoma [HCC]) (n = 10 pairs) and head and neck (HNC) (n = 6 pairs) cancers, and evaluated all-context 5mC mtDNA using the Rerio/Megalodon model validated in the previous section. HNC samples were sequenced with a MinION device, while a deeper sequencing was done in HCC samples using the high throughput version of nanopore technology, the PromethION system. Accordingly, we used the corresponding all-context neural network model for calling base modifications from each of these platforms (see “Methods”).

**Figure 7:**
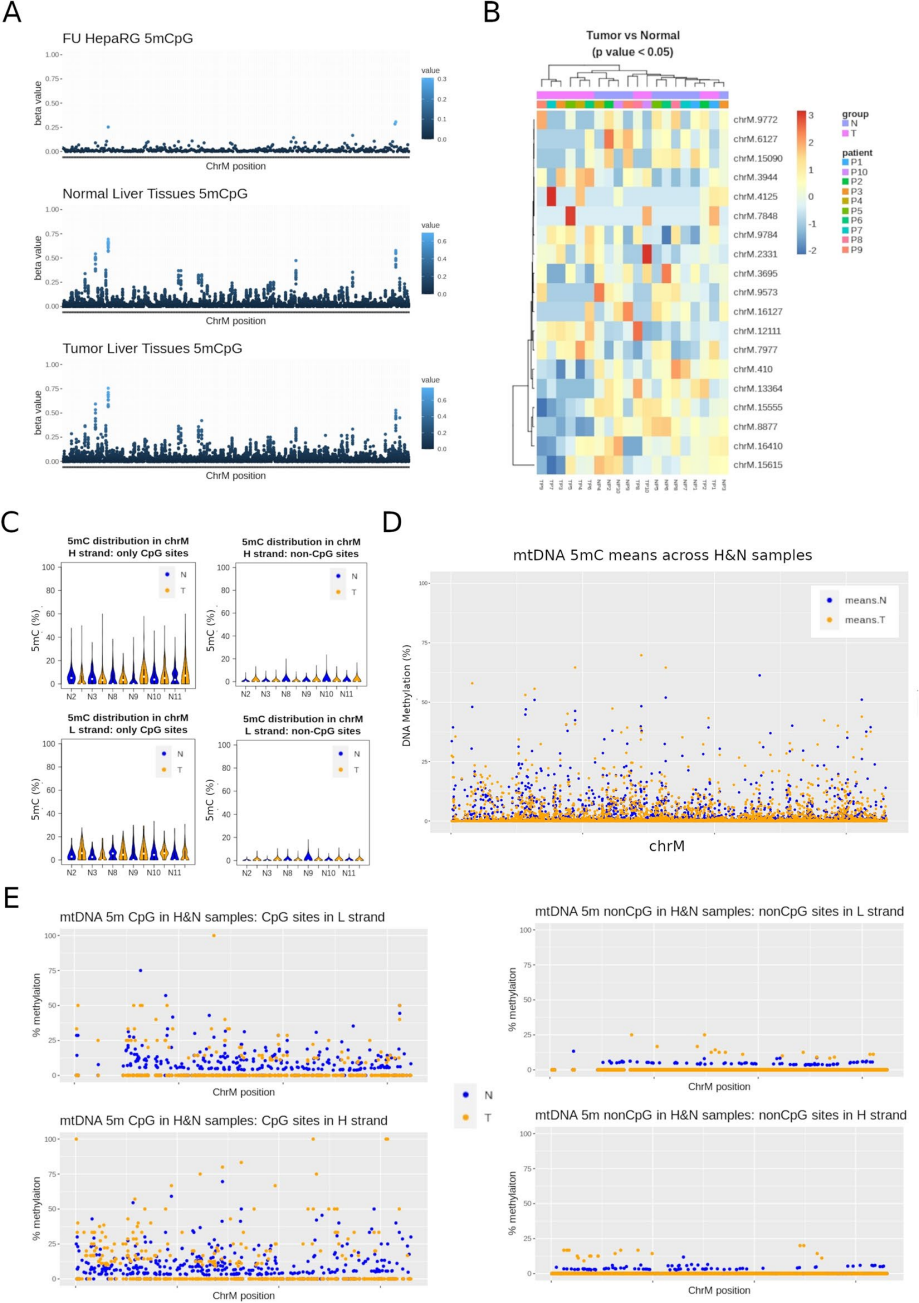
Long read DNA methylation in cancer. (**A**,**B**) DNA was extracted from 10 hepatocellular carcinoma (HCC) patients and matched non-tumor adjacent tissues. After sequencing with a PromethION device, all-context cytosine modifications were called with Megalodon. (**A**) 5mCpG values are shown after nanopore sequencing of a fully unmethylated (FU) liver cell ine (HepaRG, top panel), 10 non-tumor liver tissues (middle panel), and 10 matched tumor tissues (bottom panel). (**B**) 5mCpG heatmap of top most significant (lowest p value in the paired Tumor vs. surrounding comparison) CpG sites. Annotations include Tumor (T) vs Normal (N) status, and patient ID (P1 to P10). (**C–E**) DNA was extracted from 6 head and neck cancer (HNC) patients and matched non-tumor adjacent tissues. After sequencing with a MinON device, all-context cytosine modifications were called with Megalodon. (**C**) 5mC distribution in mtDNA represented as percentage of methylation (5mC %). Values are presented by strand (H strand in top panels, L-strand in bottom panels) and context (CpG sites on the left, CpH sites on the right). (**D**) Mean mtDNA 5mC values across all HNC tumors (means.T) and their matched adjacent tissues (means.N). All cytosines are represented. (**E**) A more stringent analysis was performed after removing low coverage sites and all reads below the length of the longest NUMT sequence (~ 8 kb). Means of HNC tumors (T) and adjacent tissues (N) are presented by strand and cytosine context separately.

In liver, both tumor and non-tumor tissues displayed 5mCpG methylation above background levels at several CpG sites (Fig. 7A). However, we did not find differentially methylated sites when comparing tumor and their matched adjacent tissues (paired, multifactor approach). A subset of CpG sites with lowest p values for this comparison (non-adjusted p < 0.05), were able to partially discriminate tumors from non-tumor tissues, with the latter displaying slightly higher levels of 5mCpG (Fig. 7B). Rather than differential methylation between tumors and non-tumor tissues, we found consistent 5mCpG at discrete sites (when compared to the FU background control) in both type of samples. Most 5mCpG was detected exclusively in the HS, and only 3 CpG sites were consistently found in the LS (i.e. chrM:314, chrM:5469, and chrM:14382). There were also more sites detected as methylated in non-tumor tissues, probably due to a higher 5mCpG variation in tumor samples (Fig. 7C). Contrary to 5mCpG, 5mCpH levels were below background and were not able to distinguish tumors from their normal counterparts.

In contrast to liver, HNC tissues displayed higher levels of both, CpG and CpH methylation. Global levels of 5mC were higher in tumor tissues (Fig. 7D), although the effect size was below 5% in all comparisons. HNC displayed higher mean methylation when taking all cytosines together (paired wilcox test p-value = 9.501e−08, mean methylation of non-tumors = 0.58%, mean methylation in tumors = 0.68%), or CpG sites (paired wilcox test p-value = 4.19e−05, mean methylation of non-tumors = 5.12%, mean methylation in tumor = 5.96%) and CpH sites (paired wilcox test p-value = 0.0002, mean methylation of non-tumors = 0.12%, mean methylation in tumors = 0.15%) independently. In addition, only site-level CpG differences between tumor and adjacent tissues reached statistical significance (Fig. 7E). Specifically, positions chrM:8582, chrM:8579, and chrM:5755 were differentially methylated in a paired tumor/adjacent analyses (FDR adjusted p value < 0.05). Such differences were found in both L- and H-strands. Of note, to avoid biases dependent on differences in coverage, we excluded cytosines with less than 10 × coverage in all samples. Furthermore, contaminating nuclear mitochondrial DNA inserts (NUMTs) are a well known source of error due to their sequence homology with mtDNA^36^. To exclude a bias due to NUMTs, we performed a separate analysis of 5mC only in nanopore reads mapping exclusively to those sequences (1030 NUMTs after liftover to the hg38 assembly, as previously described^36^). We did not find significant differences in 5mC at those locations between tumors and adjacent tissues. Moreover, in a separate analysis, we excluded all reads aligning to mtDNA with a length below 8798 bp (the longest NUMT in our reference database). After removing those reads, the global differences between tumor and non-tumor HNC tissues was still significant.

Therefore, while several cytosines were found differentially methylated in cancer tissues from HCC and HNC, we were able to detect CpH above background levels only in HNC tissues. This could be the result of tissue-dependent profiles, or the different algorithms used to call base modifications from the two devices used in these two independent datasets.

## Discussion

In the present study we have shown that nanopore sequencing can reliably detect 5mC in mitochondrial DNA from human and mouse cells. Exploiting the advantages of long reads and native DNA sequencing, we show that 5mCpG and 5mCpH can be detected at discrete locations at levels that depend on the sample (i.e. immortalized cell line versus tissues). In addition, despite global low levels, we found differential mtDNA methylation in HNC samples compared to their matched adjacent tissues. However, we did not observe differential 5mC in two biological contexts (i.e. in vitro differentiation of a liver progenitor cell line and in vitro induction of oxidative stress) or a different cancer type (HCC), where 5mC was generally low or below the threshold of detection for CpG and CpH, respectively. The absence of changes in response to oxidative stress may be specially surprising. While the effect of oxidative stress on mitochondrial activity has been extensively studied (Ashari et al., 2020; Yu et al., 2020), there had not yet been a comprehensive mapping of mtDNA 5mCpG in oxidative stress conditions. A previous study showed that downregulation of the Dnmt1 (isoform 3) under oxidative and nutritional stress, resulted in hypomethylation of mitochondrial genome. However, 5mC levels were low (close to 5% in most conditions) and only 6 selected CCGG sites were analyzed^37^. An interesting follow up of our experiment would be the study of a potential bias due to the selection of mitochondria that are being replicated after longer time points. Full mtDNA length reads would be ideal to rule out the enrichment on a particular mitochondria subpopulation.

The literature on mtDNA methylation is populated by contradictory findings that have been attributed to the limitations of bisulfite-based techniques that are considered the gold standard for base-resolution profiling of DNA methylation. In particular, it has been shown that linear mtDNA had significantly higher bisulfite conversion efficiency and resulted in lower methylation values compared with circular mtDNA^38^. In response, a series of works outlining amendments to the bisulfite conversion protocol for mitochondria have been published in order to ensure the bisulfite conversion efficiency is properly controlled for^15^. Moreover, recent publications tried to overcome such issues and conclude that mtDNA is highly methylated, in particular in a strand-specific and nonCpG context^10,35,39^. We have not been able to reproduce such findings. Indeed, absolute levels of methylation and its variation were generally low in all our samples, regardless of their origin and treatment. Although some of these discrepancies may be related to differences in sample type, this is clearly an insufficient explanation. On one hand, despite careful protocol optimization, it is difficult to fully rule out incomplete bisulfite modification due to the secondary structure of mtDNA, biases introduced by PCR amplification of two strands with a markedly different base content, or misalignments to nuclear mitochondrial sequences (i.e. NUMTs) which are more frequent with short read sequencing. On the other hand, there are limitations still present or even inherent to the novel nanopore sequencing technology. In the first case, heteroplasmy represents a challenge when identifying base-level differences in 5mC. This is especially true at the population level, but does not explain little variation in the isogenic controlled in vitro experiments shown here. In the second case, we cannot rule out that base modification calling could have been affected by local sequence patterns, such as G homopolymers. In addition, there is still room for improvement of neural network-derived models. Indeed, there is not currently a model with optimal performance which can detect 5mC, 5hmC and 6 mA at the same time. Although the accuracy of the all-context 5mC model is high, future algorithms that incorporate new base modifications simultaneously may be available in the short term, something that becomes a hindrance for bisulfite-based techniques.

We are aware of three independent studies that used nanopore sequencing to detect 5mCpG in mtDNA^40–42^. As part of a study of mitochondrial DNA alterations in oral squamous cell carcinoma, one of them evaluated the correlation between mtDNA alterations and cisplatin sensitivity using two oral squamous cell carcinoma cell lines^40^. The authors also used native DNA samples digested with BamHI to linearise the circular mitochondrial genome, followed by Nanopolish to call 5mC. Similar to our findings, they reported a low level of methylation across the whole mitochondrial genome in all tested cell lines, around 6–7%. However, the exception were three methylated sites in the H103 cell line. The authors discussed that such differences could have arisen from distinct mitochondrial and nuclear genotypes^40^. In addition, during the revision of our manuscript, two preprints were published describing the use of nanopore sequencing to detect mtDNA methylation in CpG context. One of them used a workflow similar to ours (i.e. mtDNA enrichment, linearization, and Nanopolish methylation calling) to study 5mCpG in whole blood^42^. In agreement with our findings, they detected low-level methylation, with exceptions at 32 unique CpG sites and positive correlation between both strands. Interestingly, by using synthetic DNA samples, they show that highly methylated reads had a higher tendency to be considered as failed during basecalling. Although including those reads did not dramatically change their conclusions, they rightly conclude that reanalysis of highly methylated CpG sites within mtDNA may be of importance^42^. A second preprint^41^ described a protocol also based on BamHI linearization to sequence human mtDNA. Indeed, they show that compared to fragmentation-based methods, BamHI digestion did not result in strand bias. Methylation calling using nanopolish resulted in negligible mtDNA methylation levels in multiple human primary and cancer cell lines. To avoid biases the authors recommend higher read depths that can help in the identification of heteroplasmic mtDNA variants. Indeed, they illustrate the use of de novo assembly before methylation calling to avoid such bias.

Our work is pioneer in testing all-context models of nanopore 5mC basecalling. We were able to illustrate the ability of this model to produce the expected genomic distribution of CpH in those tissues where this base modification is known to be enriched. Indeed, CpH methylation is more abundant in neurons and embryonic stem cells (ESCs), being the CAG motif most abundant in ESCs^43,44^, and the CAC motif most abundant in neurons (Guo et al., 2014; Lister et al., 2013; Xie et al., 2012). In addition, mCpHs are mostly spatially correlated with mCpGs^45–47^, a tendency that we also observed and within values closed to what has been described for brain and other tissues (i.e. below 1%)^47^. Of note, using such all-context 5mC model, we were able to find discrete differences in cancer and in particular in HNC, both at the CpG and the CpH level. Future improvements of the models used to call methylation may validate these findings while ruling out additional base modifications, a requirement for considering such changes as potential clinical biomarkers and key targets for study of their biological functions.

We provide the first comprehensive characterization of mtDNA methylation in a diversity of sample types and biological conditions, using nanopore long read sequencing. We were able to use this novel tool to detect the methylation patterns along 16 kb reads spanning the entire mitochondrial chromosome with deep coverage of > 1000 × on native DNA. In doing so, we have produced a map of mtDNA 5mC, that has eliminated any introduced bias from bisulfite conversion and PCR amplification.

## Methods

### Cell culture, maintenance and differentiation

HepaRG cells were cultured in Williams media enriched with 10% Fetal calf serum clone II, 1% Penicillin/Streptomycin, L-glutamine (2 mM), insulin (5 µg/mL) and hydrocortisone (25 µg/mL). Proliferative HepaRGs were taken before reaching 50% confluence and differentiated HepaRGs were differentiated as previously described^20,27,28^. HEK293T, immortal cells derived from embryonic kidney were grown in tissue culture dishes (Falcon, Becton Dickinson) and cultured in DMEM 1X media containing 1% Penicillin/Streptomycin, 1% sodium pyruvate, 1% L-glutamine, 1% non-essential amino-acids, all from Life Technologies, and 10% fetal bovine serum (Eurobio Abcys). Primary human hepatocytes (PHH) were isolated from healthy liver tissue as previously described^48^. DNA was extracted from liver tissue, PHH in suspension (0 h in culture) and PHH after 24 h and 7 days in culture (experimental set up outlined in Fig. 5B) using Epicentre MasterPure DNA purification kit, according to manufacturer’s instructions. In the time-point PHH experiment, DNA extraction performed with Monarch HMW DNA extraction kit for tissue (#T3060 New England Biolabs) according to manufacturers instructions. DNA was sheared using G-Tubes (Covaris) to 20 KB, 500 ng was taken forward for Rapid barcode sequencing library prep, before sequencing on a MinION device.

### Induction of oxidative stress

HEK293T cells were treated with hydrogen peroxide (H2O2) (Sigma-Aldrich, 216763) at a concentration of 500 μM for 2 h, alone or in combination with 5 mM N-acetyl-cyteine (NAC) (Sigma-Aldrich, A7250). When using NAC, cells were pre-treated for 2hrs with 5 mM NAC. The mitochondrial superoxide indicator stain MitoSOX (ThermoFisher, M36008) was used to probe the relative oxidative stress in live cells. Cells were stained with 1uM MitoSox diluted in DMEM. 250,000 cells were incubated with 330 ul for 30 min and analyzed by flow cytometry, then washed with PBS and trypsinized. Flow cytometry tubes were kept on ice and in the dark until use. Flow cytometry analysis was performed with a FACSCalibur (BD Biosciences). The mean fluorescence intensity of minimum 10,000 stained cells and unstained control cells were recorded and plotted for analysis. Alternatively, MitoSOX was analyzed by epifluorescence microscopy (Zeiss, Axio Observer).

### Tissue samples

Human biological samples and associated data were obtained from the “Tissu-Tumorothèque Est” (CRB-HCL, Hospices Civils de Lyon Biobank, BB-0033-00046) and the “Centre de Ressources Biologiques” (CRB Centre Léon Bérard, BB-0033-00050, Lyon France). This study was approved by the ethical review board of Centre Léon Bérard. The material used in the study has been collected in agreement with all applicable laws, rules, and requests of French and European government authorities, including the patient’s informed consents.

The brains of P13 OF1 mice were harvested and placed on ice-cold Dulbecco's Phosphate Buffered Saline (DPBS) for microdissection. The brain was placed on a Mouse Brain Matrix (Zivic Instruments) and coronal section of the forebrain cut at 500 µm. The dorsal subventricular zone (SVZ) was microdissected from 3 to 4 consecutive sections under a binocular microscope and collected in an Eppendorf tube containing ice-cold DPBS before being centrifuged and stored at -20 °C.

For human and mouse samples, DNA was extracted using the Epicentre MasterPure DNA purification kit, according to manufacturer’s instructions.

### Subcellular fractionation and mtDNA extraction

Subcellular fractionation was performed as previously described^49^ with some modifications. Briefly, cells were washed with PBS, harvested by scraping and centrifuged at 1000*g* for 5 min. The pellet was re-suspended in buffer containing 210 mM sorbitol, 70 mM sucrose, 1 mM EDTA, 10 mM HEPES and 0.1% BSA (Sigma) before grinding with a Dounce Homogenizer (Wheaton, USA) with a loose and tight pestle (100 strokes with each pestle). Cells were observed under microscope (Axiovert 40C, Zeiss) with trypan blue dye to assess cell membrane disruption followed by centrifugation at 500 g for 5 min at 4 °C. The supernatant was collected before centrifugation at 10 000 g for 30 min at 4 °C. DNA extraction (Nucleospin Tissue, Macherey–Nagel) was performed on the resulting pellet according to manufacturer instructions. mtDNA was digested using BamHI HF (New England BioLabs) in order to linearize the mtDNA genome.

### Fully unmethylated and fully methylated controls

After mtDNA enrichment and linearization, we prepared a negative (FU = fully unmethylated) control sample from differentiated HepaRG mtDNA by performing whole genome amplification using a repliG kit (Qiagen) according to manufacturer’s instructions. After amplification, a positive control for methylation (FM = fully methylated) was prepared. Briefly, CpG dinucleotides were methylated by incubating 1 µg of DNA with S-Adenosyl methionine (SAM) (32 µM) with CpG Methyltransferase (M.SssI) (4–25 units) (New England BioLabs) at 37 °C for 1 h before heating to 65 °C for 20mins.

### Bisulfite quantitative methyl-specific PCR (BS-qMSP)

BS-qMSP protocols were made available as detailed methods at protocols.io er^50^. Briefly, 1000 ng of each sample or standard was bisulfite converted using EZ DNA methylation Lightning kit (Zymo) according to manufacturers instructions. CpG primers were designed with MethPrimer and non-CpG primers were designed manually and purchased from Thermo Fisher Sci-entific (Waltham, MA, USA), sequences are available in supplementary table S1. Primers (0.5 µM) were combined with iTAQ Universal SYBER green Master Mix (BioRad) in 10µL reaction volumes, BS-converted DNA was amplified according to the following qPCR protocol: 95 °C 15 min, 95 °C 15seconds, 60 °C 30 s (repeat 40 ×) followed by melt curve analysis starting at 55–95°C taking measurements at 0.5 °C increments. The obtained Ct values were be used to calculate the % methylation (Beta Values) at each loci according to the following calculation:

$$\begin{gathered} 2^{ - } ({\text{CT}}\,\,{\text{values}}\,\,{\text{obtained}}\,\,{\text{for}}\,\,{\text{Methylated}}\,\,{\text{primers}}) = {\text{M}} \hfill \\ 2^{ - } ({\text{CT}}\,\,{\text{values}}\,\,{\text{obtained}}\,\,{\text{for}}\,\,{\text{Unmethylated}}\,{\text{primers}}) = {\text{U}} \hfill \\ {\text{Beta}}\,\,{\text{values}} = {\text{M}}/({\text{M}} + {\text{U}}). \hfill \\ \end{gathered}$$

### Holotomography

Differentiated and proliferative HepaRG cells were plated at high confluence. Mitotracker (100 nM) was added to normal growth medium for 1 h before imaging with a 3D Cell-Explorer Fluo (Nanolive, Ecublens, Switzerland) using a 60 × air objective. Refractory index maps were generated and images were processed every 5 s for 20 min with the STEVE software.

### Nanopore sequencing

400 ng of DNA from each sample or control was barcoded and multiplexed using the Nanopore Rapid Barcoding Sequencing kit (SQK-RBK004) according to manufacturer's instructions (Oxford Nanopore Technology). Sequencing was conducted with a Minion sequencer on ONT 1D flow cells (FLO-min106) with protein pore R9.4 1D chemistry for 48 h. Reads were basecalled with GUPPY (version 4.3.2). Basecalled reads were mapped using Minimap2 to the GRCh38/hg38 human genome. Liver cancer (HCC) and matched non-tumor samples were barcoded with the Ligation Sequencing Kit (SQK-LSK109) and sequenced with a PromethION device.

### Bioinformatic analyses

Our analyses are divided in two parts, our initial CpG methylation pipeline based on running Nanopolish in parallel to Guppy + Medaka for each sample, and the second one based on Megalodon to call 5mC in all contexts (i.e. CpG and CpH).

For our initial CpG methylation analyses, basecalling was performed with Guppy version 4.0.15 (ONT). We first determined the methylation status of each CpG site on every read by using the widely used tool, *nanopolish*^23^ used recently by^51^. For validation, we also called DNA methylation using the novel tool, Medaka (https://github.com/nanoporetech/medaka). Medaka is a tool to create a consensus sequence from nanopore sequencing data. This task is performed using neural networks applied from a pileup of individual sequencing reads against a draft assembly. It outperforms graph-based methods operating on basecalled data, and can be competitive with state-of-the-art signal-based methods, whilst being much faster. Both tools have been recently benchmarked^22^. PycoQC was used for data inspection and quality control (https://github.com/a-slide/pycoQC), and methplotlib (https://github.com/wdecoster/methplotlib) for read-level visualizations. Called CpG sites in the FU control were used to determine a baseline of methylation. The following calculation was utilised: FalsePositiveRate = [#called methylated cytosines in FU/#called cytosines in FU].

To call 5mC in all contexts, we first demultiplexed the reads at the raw (fast5) level using Deepbinner (v.0.2.0)^52^, before basecalling and extracting base modification information using the most recent model from the Rerio repository (https://github.com/nanoporetech/rerio) implemented through the Megalodon tool (v.2.2.9) (https://github.com/nanoporetech/megalodon). Megalodon extracts high accuracy modified base and sequence variant calls from raw nanopore reads by anchoring the information rich basecalling neural network output to a reference genome. Specifically, we used the corresponding MinION (res_dna_r941_min_modbases_5mC_v001.cfg v.4.2.2) and PromethION (res_dna_r941_prom_modbases_5mC_v001.cfg v.4.2.2) models, with a –mod-binary-threshold of 0.8, as recommended. To validate CpH analyses, we used Megalodon (as described above) to re-basecall raw fast5 data from a published dataset^53^ (https://www.ebi.ac.uk/ena/browser/view/PRJEB33258). As these libraries were prepared after cas9-targeting of selected genes (3 gRNA designs, that resulted in 19 hg38 genomic regions), we pooled the reads from several samples to be able to plot as enriched heatmaps (Fig. 6D,E).

For differential methylation analyses we used DSS (Dispersion shrinkage for sequencing data)^54^ adapted for nanopore sequencing^51^. Briefly, DSS tests for differential methylation at single CpG-sites, using a Wald test on the coefficients of a beta-binomial regression of count data with an ‘arcsine’ link function. In order to set minimum requirements for DSS analysis, an internal comparison of biological replicates of differentiated HepaRG cells was undertaken. From this we were able to better understand the background and determine the minimum smoothing and delta values. These values were set at a delta of 0.05 with minimum P-value of 0.05. For transcription factor binding site analyses, we used the bioconductor packages MIRA^21^ for methylation data aggregation, and LOLA for dataset selection^55^. Mann–Whitney’s test was used for pairwise comparisons of 5mCpG distribution.

## Supplementary Information

Supplementary Information

## Data Availability

Datasets generated during the current study have been uploaded to the GEO repository (fastq files and medaka-extracted methylation tables have been submitted under accession #GSE158224 and # GSE159582; submitter: H Hernandez-Vargas).

## References

[1] Porporato PE, Filigheddu N, Pedro JMB-S, Kroemer G, Galluzzi L (2018). Mitochondrial metabolism and cancer. Cell Res..

[2] Alberts, B. *et al. Molecular biology of the cell*. (Garland Science, 2002).

[3] Alexeyev, M., Shokolenko, I., Wilson, G. & LeDoux, S. The maintenance of mitochondrial DNA integrity—critical analysis and update. *Cold Spring Harb. Perspect. Biol.* **5**, (2013). 10.1101/cshperspect.a012641 PMC3632056 23637283

[4] Sun X, St John JC (2018). Modulation of mitochondrial DNA copy number in a model of glioblastoma induces changes to DNA methylation and gene expression of the nuclear genome in tumours. Epigenetics Chromatin.

[5] Weinhouse C (2017). Mitochondrial-epigenetic crosstalk in environmental toxicology. Toxicology.

[6] Lieber CS (1991). Hepatic, metabolic and toxic effects of ethanol: 1991 update. Alcohol. Clin. Exp. Res..

[7] Bellizzi D (2013). The control region of mitochondrial DNA shows an unusual CpG and non-CpG methylation pattern. DNA Res..

[8] Crews S, Ojala D, Posakony J, Nishiguchi J, Attardi G (1979). Nucleotide sequence of a region of human mitochondrial DNA containing the precisely identified origin of replication. Nature.

[9] Fish J, Raule N, Attardi G (2004). Discovery of a major D-loop replication origin reveals two modes of human mtDNA synthesis. Science.

[10] Dou X (2019). The strand-biased mitochondrial DNA methylome and its regulation by DNMT3A. Genome Res..

[11] Feng S, Xiong L, Ji Z, Cheng W, Yang H (2012). Correlation between increased ND2 expression and demethylated displacement loop of mtDNA in colorectal cancer. Mol. Med. Rep..

[12] Pirola CJ (2013). Epigenetic modification of liver mitochondrial DNA is associated with histological severity of nonalcoholic fatty liver disease. Gut.

[13] Hong EE, Okitsu CY, Smith AD, Hsieh C-L (2013). Regionally specific and genome-wide analyses conclusively demonstrate the absence of CpG methylation in human mitochondrial DNA. Mol. Cell. Biol..

[14] Mechta M, Ingerslev LR, Fabre O, Picard M, Barrès R (2017). Evidence suggesting absence of mitochondrial DNA methylation. Front Genet.

[15] Owa C, Poulin M, Yan L, Shioda T (2018). Technical adequacy of bisulfite sequencing and pyrosequencing for detection of mitochondrial DNA methylation: Sources and avoidance of false-positive detection. PLoS ONE.

[16] Li Y, Tollefsbol TO (2011). DNA methylation detection: bisulfite genomic sequencing analysis. Methods Mol. Biol..

[17] Madoui M-A (2015). Genome assembly using Nanopore-guided long and error-free DNA reads. BMC Genomics.

[18] Seki M (2019). Evaluation and application of RNA-Seq by MinION. DNA Res..

[19] Jain M, Olsen HE, Paten B, Akeson M (2016). The Oxford Nanopore MinION: Delivery of nanopore sequencing to the genomics community. Genome Biol..

[20] Rodríguez-Aguilera JR (2020). Genome-wide 5-hydroxymethylcytosine (5hmC) emerges at early stage of in vitro differentiation of a putative hepatocyte progenitor. Sci. Rep..

[21] Lawson JT, Tomazou EM, Bock C, Sheffield NC (2018). MIRA: an R package for DNA methylation-based inference of regulatory activity. Bioinformatics.

[22] Yuen, Z. W.-S, et al. Systematic benchmarking of tools for CpG methylation detection from Nanopore sequencing. *bioRxiv* 2020.10.14.340315 (2020). 10.1101/2020.10.14.340315. 10.1038/s41467-021-23778-6 PMC8187371 34103501

[23] Simpson JT (2017). Detecting DNA cytosine methylation using nanopore sequencing. Nat. Methods.

[24] Wick RR, Judd LM, Holt KE (2019). Performance of neural network basecalling tools for Oxford Nanopore sequencing. Genome Biol..

[25] Gilpatrick T (2020). Targeted nanopore sequencing with Cas9-guided adapter ligation. Nat. Biotechnol..

[26] Yu Y (2012). Hepatocyte-like cells differentiated from human induced pluripotent stem cells: Relevance to cellular therapies. Stem Cell Res..

[27] Ancey P-B (2017). TET-catalyzed 5-hydroxymethylation precedes HNF4A promoter choice during differentiation of bipotent liver progenitors. Stem Cell Rep..

[28] Cerec V (2007). Transdifferentiation of hepatocyte-like cells from the human hepatoma HepaRG cell line through bipotent progenitor. Hepatology.

[29] Yagi H, Tan J, Tuan RS (2013). Polyphenols suppress hydrogen peroxide-induced oxidative stress in human bone-marrow derived mesenchymal stem cells. J. Cell. Biochem..

[30] Nestor CE (2015). Rapid reprogramming of epigenetic and transcriptional profiles in mammalian culture systems. Genome Biol..

[31] Guo JU (2014). Distribution, recognition and regulation of non-CpG methylation in the adult mammalian brain. Nat. Neurosci..

[32] Lister R (2013). Global epigenomic reconfiguration during mammalian brain development. Science.

[33] Xie W (2012). Base-resolution analyses of sequence and parent-of-origin dependent DNA methylation in the mouse genome. Cell.

[34] the metabolic requirements of cell proliferation (2009). Vander Heiden, M. G., Cantley, L. C. & Thompson, C. B. Understanding the Warburg effect. Science.

[35] Patil V (2019). Human mitochondrial DNA is extensively methylated in a non-CpG context. Nucleic Acids Res..

[36] Li M, Schroeder R, Ko A, Stoneking M (2012). Fidelity of capture-enrichment for mtDNA genome sequencing: Influence of NUMTs. Nucleic Acids Res..

[37] Saini SK, Mangalhara KC, Prakasam G, Bamezai RNK (2017). DNA Methyltransferase1 (DNMT1) Isoform3 methylates mitochondrial genome and modulates its biology. Sci. Rep..

[38] Liu B (2016). CpG methylation patterns of human mitochondrial DNA. Sci. Rep..

[39] Ghosh S, Sengupta S, Scaria V (2014). Comparative analysis of human mitochondrial methylomes shows distinct patterns of epigenetic regulation in mitochondria. Mitochondrion.

[40] Aminuddin A, Ng PY, Leong C-O, Chua EW (2020). Mitochondrial DNA alterations may influence the cisplatin responsiveness of oral squamous cell carcinoma. Sci. Rep..

[41] Bicci, I., Calabrese, C., Golder, Z. J., Gomez-Duran, A. & Chinnery, P. F. Oxford Nanopore sequencing-based protocol to detect CpG methylation in human mitochondrial DNA. *bioRxiv* 2021.02.20.432086 (2021). 10.1101/2021.02.20.432086.

[42] Lüth, T. *et al.* Analysis of mitochondrial genome methylation using Nanopore single-molecule sequencing. *bioRxiv* 2021.02.05.429923 (2021) 10.1101/2021.02.05.429923.

[43] Laurent L (2010). Dynamic changes in the human methylome during differentiation. Genome Res..

[44] Lister R (2009). Human DNA methylomes at base resolution show widespread epigenomic differences. Nature.

[45] Lee, J.-H., Saito, Y., Park, S.-J. & Nakai, K. Existence and possible roles of independent non-CpG methylation in the mammalian brain. *DNA Res.* **27**, (2020). 10.1093/dnares/dsaa020 PMC7750974 32970817

[46] Lee J-H, Park S-J, Nakai K (2017). Differential landscape of non-CpG methylation in embryonic stem cells and neurons caused by DNMT3s. Sci Rep.

[47] Ziller MJ (2011). Genomic distribution and inter-sample variation of non-CpG methylation across human cell types. PLoS Genet..

[48] Lecluyse EL, Alexandre E (2010). Isolation and culture of primary hepatocytes from resected human liver tissue. Methods Mol. Biol..

[49] Arnoult D (2003). Mitochondrial release of AIF and EndoG requires caspase activation downstream of Bax/Bak-mediated permeabilization. EMBO J..

[50] Hernandez-Vargas H, Goldsmith C (2020). Quantitative analysis of methylation and hydroxymethylation using oXBS-qMSP..

[51] Gigante S (2019). Using long-read sequencing to detect imprinted DNA methylation. Nucleic Acids Res..

[52] Wick RR, Judd LM, Holt KE (2018). Deepbinner: Demultiplexing barcoded Oxford Nanopore reads with deep convolutional neural networks. PLoS Comput. Biol..

[53] Wongsurawat T (2020). A novel Cas9-targeted long-read assay for simultaneous detection of IDH1/2 mutations and clinically relevant MGMT methylation in fresh biopsies of diffuse glioma. Acta Neuropathol Commun.

[54] Park Y, Wu H (2016). Differential methylation analysis for BS-seq data under general experimental design. Bioinformatics.

[55] Sheffield NC, Bock C (2016). LOLA: enrichment analysis for genomic region sets and regulatory elements in R and Bioconductor. Bioinformatics.

