# Supplementary material for: Low biological fluctuation of mitochondrial CpG and non-CpG methylation at the single-molecule level
Source: Sci Rep. 2021 Apr 13;11:8032. doi: 10.1038/s41598-021-87457-8 (PMC8044111; doi:10.1038/s41598-021-87457-8)
Supplement: Supplementary file 1 — Supplementary Information [file 41598_2021_87457_MOESM1_ESM.pdf]

Low biological fluctuation of mitochondrial CpG and non-CpG methylation at the single-molecule level

Chloe Goldsmith[1]*, Jesús Rafael Rodríguez-Aguilera[2], Ines El-Rifai[1], Adrien Jarretier-Yuste[1], Valérie Hervieu[3], Olivier Raineteau[4], Pierre Saintigny[5,6], Victoria Chagoya de Sánchez[2], Robert Dante[7], Gabriel Ichim[8] and Hector Hernandez-Vargas[1,6]*

Affiliations:

1. Department of Tumor Escape, Resistance and Immunity. TGF-beta and immuno-regulation Team. Cancer Research Centre of Lyon (CRCL), INSERM U 1052, CNRS UMR 5286, Université de Lyon, Centre Léon Bérard, 28 rue Laennec, 69373 Lyon CEDEX 08, France.
2. Department of Cellular Biology and Development, Instituto de Fisiología Celular, Universidad Nacional Autónoma de México (UNAM), Circuito Exterior s/n, Ciudad Universitaria, Coyoacán 04510, Cd. Mx., Mexico.
3. Department of Surgical Pathology, Hospices Civils de Lyon, Groupement Hospitalier Est, Lyon, France.
4. Univ Lyon, Université Claude Bernard Lyon 1, INSERM, Stem Cell and Brain Research Institute U1208, Bron, France.
5. Univ Lyon, Université Claude Bernard Lyon 1, INSERM 1052, CNRS 5286, Centre Léon Bérard, Centre de Recherche en Cancérologie de Lyon, Lyon, France ;
6. Department of Translational Medicine, Centre Léon Bérard, Lyon, France;
7. Dependence Receptors Cancer and Development Laboratory, Department of Signaling of Tumoral Escape. Cancer Research. Center of Lyon (CRCL), Inserm U 1052, CNRS UMR 5286, Université de Lyon, Centre Léon Bérard, 28 rue Laennec, 69373 Lyon CEDEX 08, France.
8. Cancer Cell Death Laboratory, Part of LabEx DEVweCAN, Université de Lyon, Lyon, France. Cancer Research Centre of Lyon (CRCL), Inserm U 1052, CNRS UMR 5286, Université de Lyon, Centre Léon Bérard, 28 rue Laennec, 69373 Lyon CEDEX 08, France.

*** Corresponding Authors**

chloe.goldsmith@inserm.fr ; hector.hernandez-vargas@lyon.unicancer.fr

Department of Tumor Escape, Resistance and Immunity. TGF-beta and immuno-regulation Team. Cancer Research Centre of Lyon (CRCL), INSERM U 1052, CNRS UMR 5286, UCBL1. Centre Léon Bérard, 28 rue Laennec, 69373, Lyon, CEDEX 08, France.

Supplementary Information

Supplementary Figure 1. Supplement to Figure 2. Quality control of mtDNA enrichment after subcellular fractionation and mtDNA extraction. Original images obtained after mtDNA enrichment in two different cell lines (i.e. HeLa and 293T) using western blot against b-Tubulin, GAPDH, and COX-IV in mitochondrial (M) and cytosolic (C) fractions. The blot was scanned after short (A) and long (B) exposure.

Supplementary Figure 2. Technical validation. A) Methylation of mtDNA in Fully methylated (FM) and Unmethylated (FU) controls by two techniques, Nanopore sequencing (Nanopore) and Bisulfite quantitative methyl-specific PCR (BS-qMSP). B) mtDNA methylation of heavy strand (H) and light strand (L) of samples determined by BS-qMSP separated into groups of in vitro immortalized cells (Cells), Primary cultured human hepatocytes (PHH) and Liver tissue (Tissue). C) mtDNA CpG methylation (CG) and non-CpG methylation (CH) of samples determined by BS-qMSP separated into groups of in vitro immortalized cells (Cells), Primary cultured human hepatocytes (PHH) and Liver tissue (Tissue). D) mtDNA methylation of heavy strand (H) and light strand (L) of liver tissue samples determined by BS-qMSP separated into Tumor tissue (T) and Normal tissue (N) matched pairs. E) mtDNA CpG methylation (CG) and non-CpG methylation (CH) of Liver tissue samples determined by BS-qMSP separated into Tumor tissue (T) and Normal tissue (N) matched pairs. Plots B to D combine the results of several CpG sites (3x H-strand and 4x L-strand), non-CpG sites (H strand, 2 x L-strand), and sample replicates (PHH: n=4, Cells: n=8, Tissue: n=20).

Supplementary Table 1. List of primers used for validation with bisulfite quantitative methyl-specific PCR (qMSP).

Supplement to Figure 2

A

Exposure 1

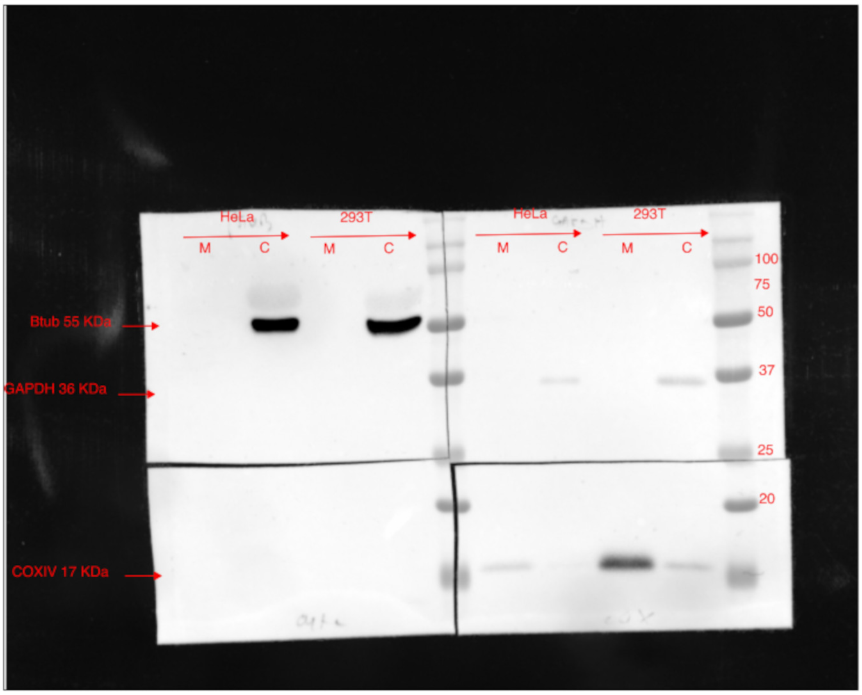

B

Exposure 2

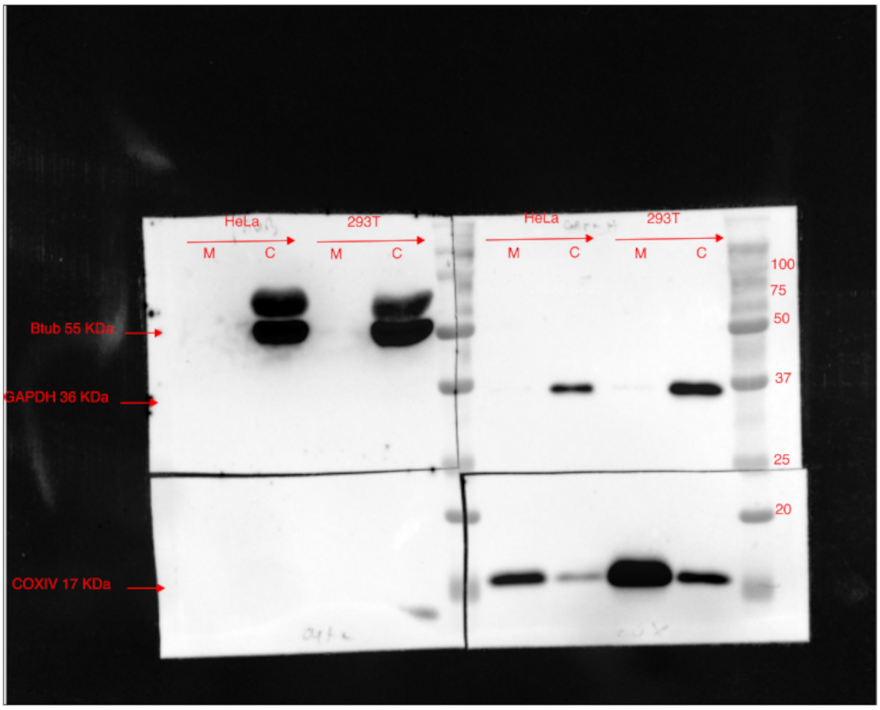

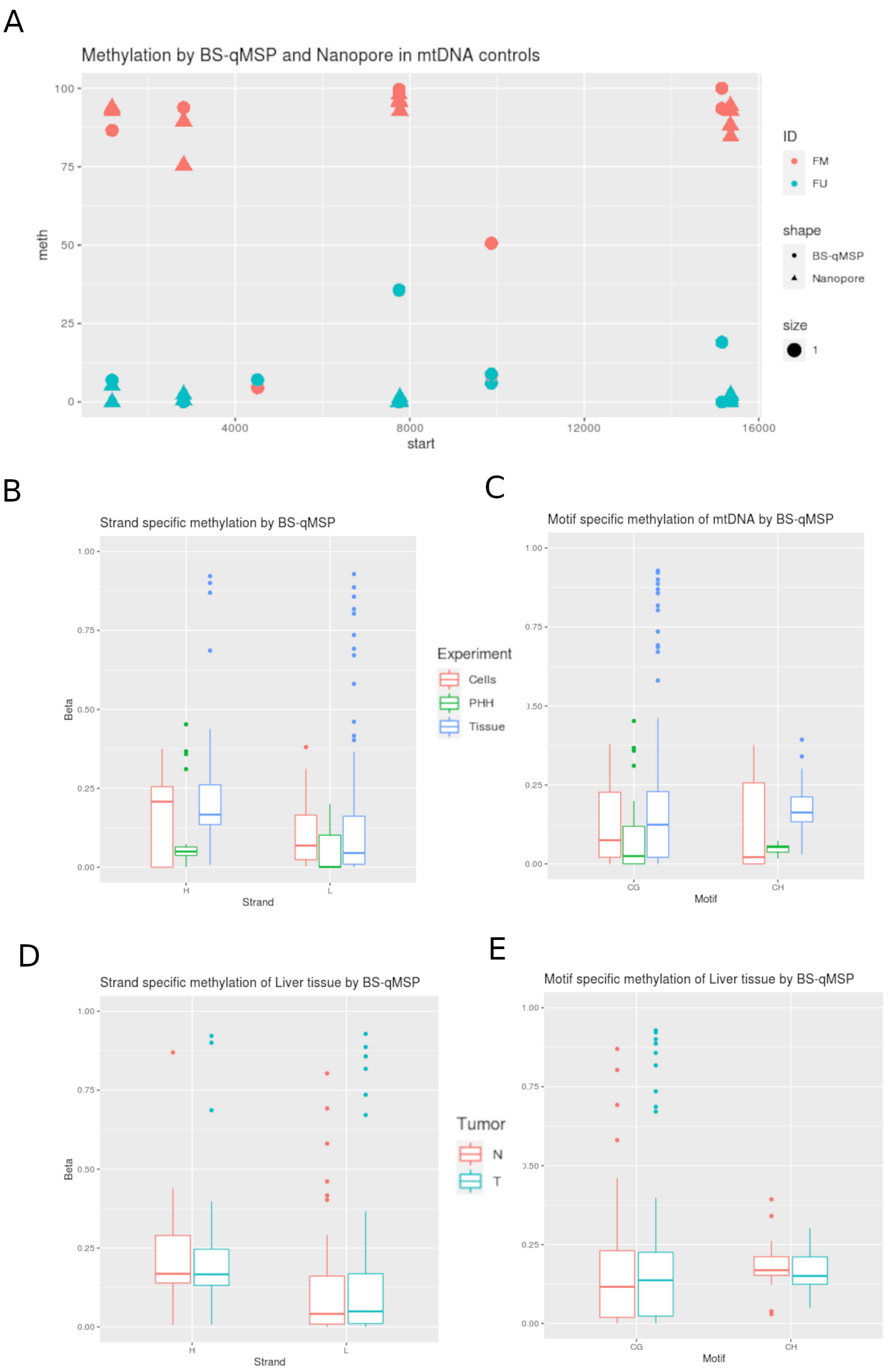

Supplementary Figure 2

Supplementary Table 1.

Oligo sequence (5' to 3')	Oligo name
TATGTAGGAGTTGAAGATTA	H_CpG_mtDNA_MF3
TCAAACGCTCAAAAAATAAAAACCGTCT	H_CpG_mtDNA_MR3
GTAGGAGTTGAAGATTAGTTTGTTG	H_CpG_mtDNA_UF3
CTCAAACACTCAAAAAATAAAAACCA	H_CpG_mtDNA_UR3
GGTTGGTTAGGGTATAATTGTTTGG	H_CpG_mtDNA_MF4
CCTCCTATTCTTACACGAAACG	H_CpG_mtDNA_MR4
GGTTGGTTAGGGTATAATTGTTTGGGTTG	H_CpG_mtDNA_UF4
CCTCCTATTCTTACACAAAACAAAA	H_CpG_mtDNA_UR4
TAAAATTTAAAGGATTTGGCGG	L_CPG_mtDNA_MF1
ATAAACTACACCTTAACCTAACGTC	L_CpG_mtDNA_MR1
TAAAATTTAAAGGATTTGGTGG	L_CpG_mtDNA_UF1
ACCTCATAAACTACACCTTAACCTAACAT	L_CpG_mtDNA_UR1
GCGGGTATAATATAGTAAGACGAGA	L_CpG_mtDNA_MF2
AATTAAATTCTACTCCGAAATCGC	L_CpG_mtDNA_MR2
GGTGGGTATAATATAGTAAGATGAGA	L_CpG_mtDNA_UF2
AATTAAATTCTACTCCAAAATCACCC	L_CpG_mtDNA_UR2
TTTAGACGTTTAGGAAATAGAAATCG	L_CpG_mtDNA_MF3
ATAAAAATTAAAAATTAATCCGCCG	L_CpG_mtDNA_MR3
TAGATGTTTAGGAAATAGAAATTG	L_CpG_mtDNA_UF3
TATATAAAAATTAAAAATTAATCCACCA	L_CpG_mtDNA_UR3
TTTATTTTTGTACGAAACGGGA	L_CpG_mtDNA_MF4
TAACTAAAATATAATTATCTAAATCGCC	L_CpG_mtDNA_MR4
TATTTTTGTATGAAATGGGA	L_CpG_mtDNA_UF4
AACTAAAATATAATTATCTAAATCACCT	L_CpG_mtDNA_UR4
TAGTTAATTGGAAGTTAAtgGTA	H_CG_Unmeth_a5_Fwd
TAGTTAATTGGAAGTTAAcgGTA	H_CG_Meth_a5_Fwd
AATTATTAGTAGTAAGGtTAGGA	H_CH_Unmeth_a5_Fwd
AATTATTAGTAGTAAGGcTAGGA	H_CH_Meth_a5_Fwd
ACTAATATTTCACTTTACATCCA	H_a5_Rev
ATGGTAAGTGTAtTGGAAAGT	L_CH_Unmeth_a1_Fwd
ATGGTAAGTGTAcTGGAAAGT	L_CH_Meth_a1_Fwd
TTATAGAAATTTAGGTTAAATA	L_a2_Fwd
AATAGTGGGAAGATTTATAGG	L_a1_Rev
CCTCTTTTTACCAACTC	L_CH_Unmeth_a2_Rev
CCTCTTTTTACCAGCTC	L_CH_Meth_a2_Rev
TGGTTAGAAtTGGAATAAAAG	H_CH_Unmeth_a4_Fwd
TGGTTAGAAcTGGAATAAAAG	H_CH_Meth_a4_Fwd
TCATCTACTCTACCATCTTT	H_a4_Rev
